# Phloem sap in Cretaceous ambers as abundant double emulsions preserving organic and inorganic residues

**DOI:** 10.1038/s41598-020-66631-4

**Published:** 2020-06-16

**Authors:** Rafael Pablo Lozano, Ricardo Pérez-de la Fuente, Eduardo Barrón, Ana Rodrigo, José Luis Viejo, Enrique Peñalver

**Affiliations:** 1 Museo Geominero-Instituto Geológico y Minero de España, Ríos Rosas 23, Madrid, 28003 Spain; 2 Oxford University Museum of Natural History, Parks Road, Oxford, OX1 3PW UK; 3 Facultad de Ciencias, Universidad Autónoma de Madrid, Darwin 2, 28049 Madrid, Spain

**Keywords:** Plant evolution, Biogeochemistry, Palaeontology

## Abstract

Fossilized remains preserved in amber provide abundant data on the paleobiota surrounding the resin-producing plants, but relatively scarcer information about the resinous sources themselves. Here, dark pseudoinclusions in kidney-shaped amber pieces from the Early Cretaceous (Albian) amber from Spain are studied. This type of fossilized remain, abundant in Cretaceous ambers, was first interpreted as fossilized vacuole-bearing microorganisms, but later regarded as artifactual and probably secreted by the resinous trees, although their origin remained unclear. Using complementary microscopy (light, electron, confocal), spectroscopy (infrared, micro-Raman), mass spectrometry and elemental analysis techniques, we demonstrate that the pseudoinclusions correspond to droplets of phloem sap containing amber spheroids and preserving both organic and inorganic residues consistent with degraded components from the original sap. The amber pieces containing pseudoinclusions are fossilized, resin-in-sap-in-resin double emulsions, showing banding patterns with differential content of resin-in-sap emulsion droplets. Our findings represent the first time fossilized phloem sap, 105 million years old, has been recognized and characterized, and open new lines of paleontological research with taxonomic, taphonomic, physiological and ecological implications.

## Introduction

Amber, fossilized plant resin, is found from the Carboniferous through to pre-Quaternary times^1,2^. A wide range of biotic and abiotic components characteristic of the ancient forests became entrapped in sticky resin as inclusions^3,4^. Due to the exceptional conservation of biological remains that amber provides, research efforts have been hitherto focused on the biological inclusions, typically macroscopic arthropods such as insects and arachnids. These bioinclusions not only provide valuable taxonomic/phylogenetic information, but also remarkable paleoecological, paleogeographical and paleobehavioral data^1–3^. Microscopic amber inclusions, on the other hand, have been scarcely studied in comparison. Although microorganisms are known to have the potential for remarkable preservation in fossil resins, and a promising diversity of such organisms is known from Cretaceous amber (e.g.^5,6^), dark inclusions with a vacuolated appearance first described as fossilized vacuole-bearing microorganisms (e.g.^7–11^) were later regarded as non-microbial in origin, and termed pseudo-protists or pseudoinclusions^12–16^. The microbiological nature of these pseudoinclusions was first questioned on the basis of size range, spatial distribution, preferred orientation, and lack of surface ornamentation or inner structures, such as nuclei or organelles, based on material from mid-Cretaceous French amber^12–14^. These pseudoinclusions were regarded as being either secreted by the resiniferous tree itself or as a product of the diagenetic polymerization of resin^13^. Similar pseudoinclusions from Australian amber were also deemed “structural artifacts created during the exudation of the original resin”^15^. More recently, ToF-SIMS analyses on pseudoinclusions “comprising aqueous soluble compounds that have been immiscible with the hydrophobic, terpenoid-based resin matrix” within extant *Agathis* resin showed that these inclusions were likely secreted together with the resin, and supported their non-microbiological origin^16^. In the latter study, the authors concluded that the pseudoinclusions were (1) secreted by the epithelial cells along the resin canals or blisters, (2) derived from the resin-synthesizing vacuoles, or (3) originated “from substances within the tree sap, which could have been stirred within the resin flow”. Strikingly, the latter hypothesis had been already put forward at the end of the 19^th^ century, based on examinations of Burmese amber: “These bodies [small, rounded, elongated objects whose color is more or less dark brown] obviously originate from sap that was excreted along, and simultaneously, with the resin from the plant stem”^16,17^. Whereas pseudoinclusions are known to frequently occur in diverse Cretaceous ambers, they are also present in Cenozoic amber and in modern resins^13,16,18^, for example in *Hymenaea verrucosa* resin from Madagascar (own observations; E.P.).

The Rábago/El Soplao amber outcrop (Cantabria, Spain) occurs within an Early Cretaceous (Albian) siliciclastic unit, more particularly associated with silty sandstones and carbonaceous claystones deposited in an interdistributary bay^19^. Chemical analyses suggest that the conifer families Cupressaceae, Podocarpaceae and Cheirolepidiaceae produced the resin that originated the amber deposit^20^, and pollen grains from these groups are present in the amber-bearing sediments^21^. Although a few amber pieces in the outcrop are stalactite-shaped, resulting from successive resin flows secreted under aerial conditions, and which are often highly fossiliferous, most of the amber occurs as large, kidney-shaped pieces, up to 25 cm length^19,22^. These pieces lack plant or animal bioinclusions, as the resin emission likely took place in confined, unexposed (underground or in trunk pockets) conditions^19,22^. Dark-light banding patterns were reported in amber; although of unclear origin, they were attributed to bubbles or other inorganic or organic matter content^23–25^.

In this study, we use morphological, taphonomic and chemical evidence obtained from microscopic, spectroscopic, mass and elemental analyses to prove that the amber pieces bearing abundant pseudoinclusions in the Rábago/El Soplao amber, which are also common in other Cretaceous ambers, are composed of fossilized phloem sap and resin that originally mixed in double emulsion, as both immiscible liquids were extruded together. Kidney-shaped amber pieces are ideal to study pseudoinclusions, since their content in the latter is higher than that of other amber types and they are more suitable for destructive preparation due to their lack of valuable macroscopic bioinclusions.

## Results

### Morphological and distributional data

**Figure 1:**
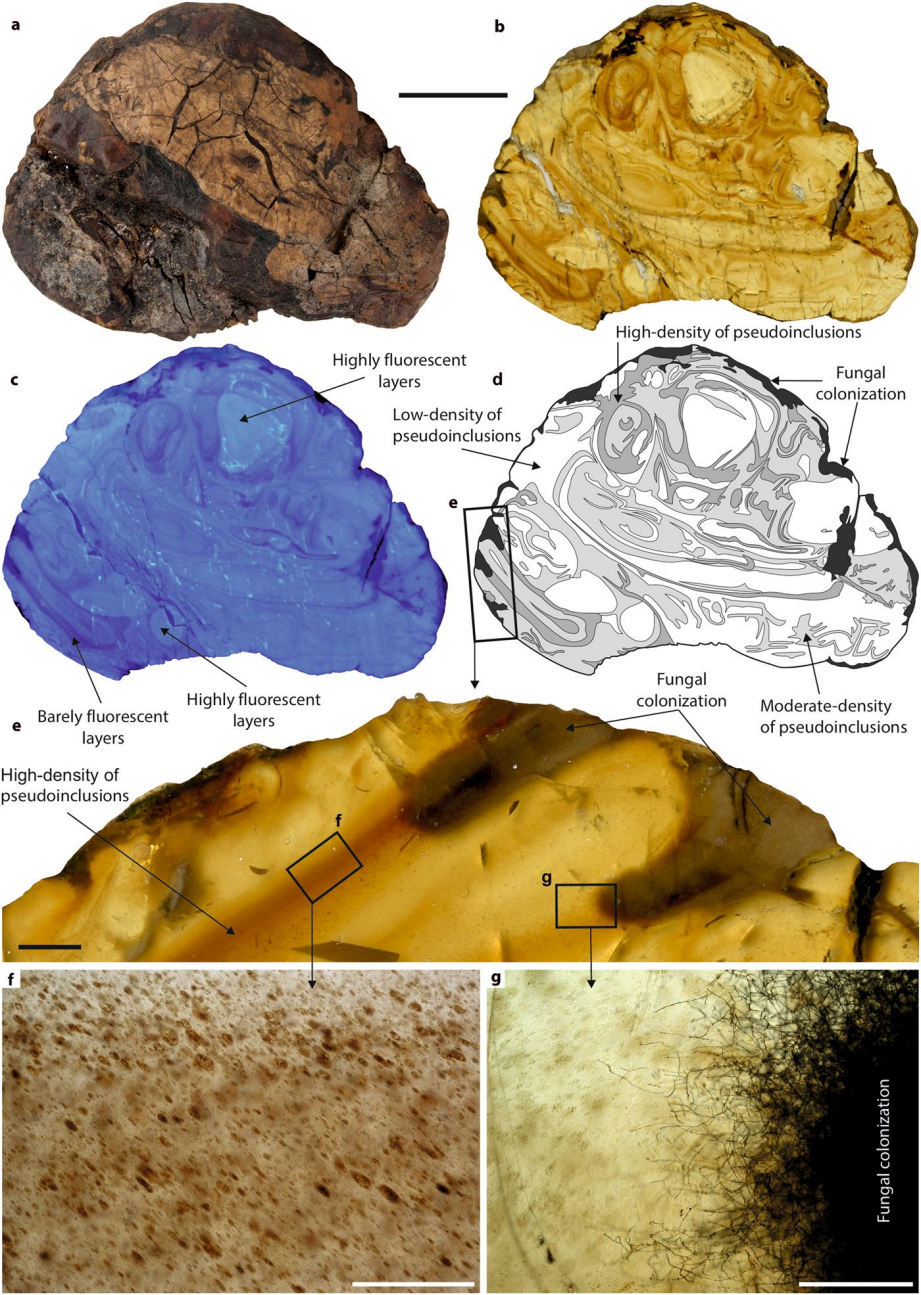
Banding pattern in a kidney-shaped amber piece from Rábago/El Soplao amber and fungal colonization. (**a**) Surface appearance of the piece. (**b**) Polished section of the same piece (1 mm thick; preparation 18067). Note the areas where the layers with high and low density of pseudoinclusions are evident. (**c**) Long-wave ultraviolet light image of the same section. The darker (less fluorescent) layers are the richest in pseudoinclusions and thus richest in fossilized phloem sap. (**d**) Drawing of the same amber piece, showing layers with low, moderate, and high concentration of pseudoinclusions (white, light grey and dark grey, respectively) and fungal colonization (black). (**e**) Magnified inset in (**d**), showing how fungal colonization took place preferentially along resin layers richest in pseudoinclusions (richest in fossilized phloem sap). (**f**) Detail of pseudoinclusion-rich amber layer shown in (**e**). (**g**) Front of advance of a fungal colonization (mycelium) shown in (**e**). Scale bars: 3 cm (**a–d**), 2 mm (**e**), 0.5 mm (**f–g**). Illustration created using CorelDRAW Graphics Suite X8 (www.coreldraw.com).

**Figure 2:**
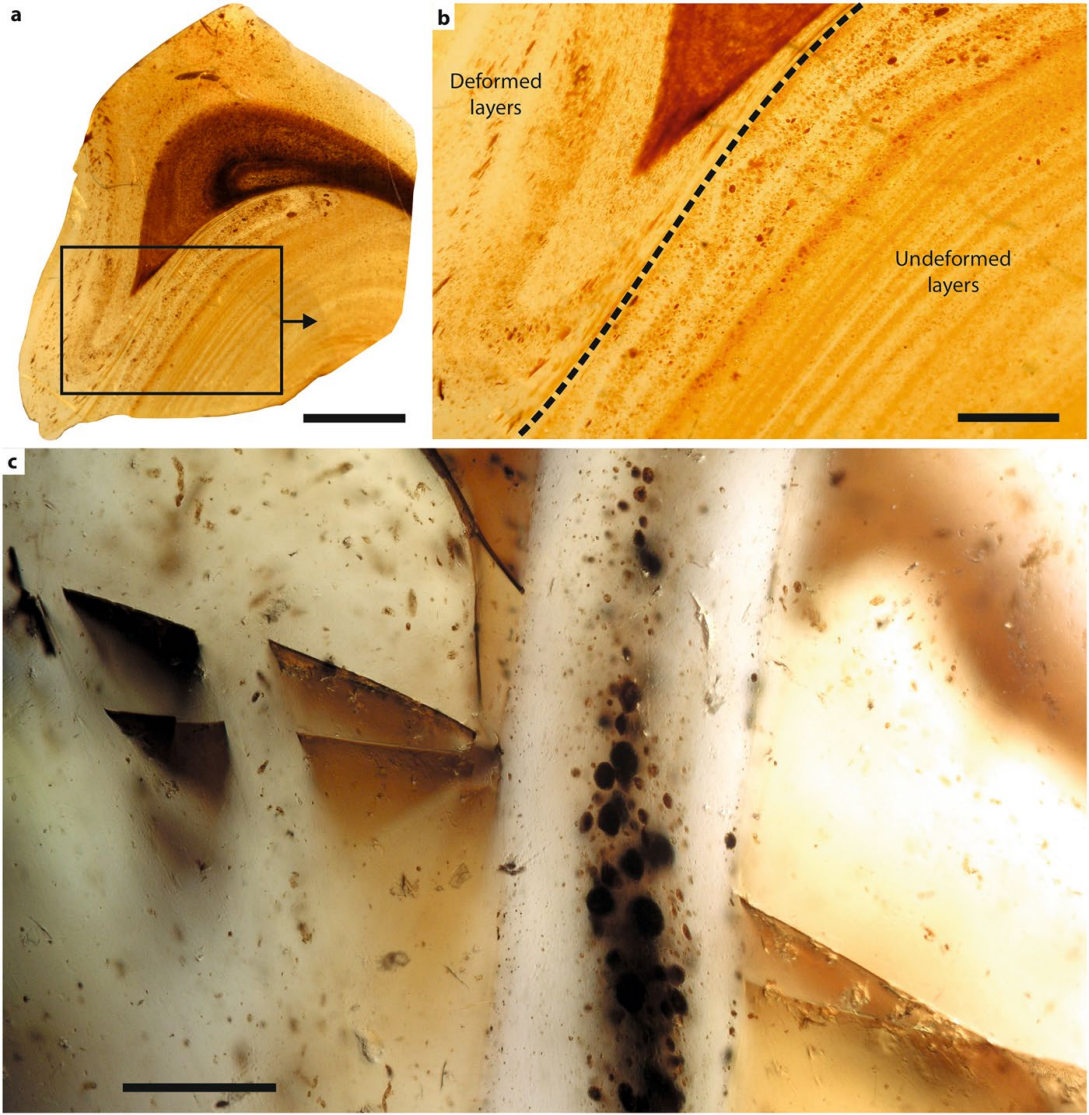
Layers of pseudoinclusions in kidney-shaped amber pieces. (**a**) Amber section where deformed and undeformed layers are found together (from preparation 18068). (**b**) Detail of the contact between deformed and undeformed amber layers in (**a**), showing deformed and undeformed pseudoinclusions, respectively. (**c**) Layer rich in pseudoinclusions (at the middle of the photograph) cutting off previous layers (or resin inputs) (from preparation 18070). Scale bars: 3 mm (**a**), 1 mm (**b**), 300 µm (**c**). Illustration created using CorelDRAW Graphics Suite X8 (www.coreldraw.com).

The kidney-shaped amber pieces from the Rábago/El Soplao outcrop show an alternation between light and dark layers (Fig. 1a,b). The dark layers contain a high density of pseudoinclusions, whereas the light layers contain lower densities (Fig. 1d,e). This banding pattern is also apparent under long-wave ultraviolet light as a gradation in the autofluorescence intensity between the dark, pseudoinclusion-rich layers (barely fluorescent) and the light, pseudoinclusion-poor layers (highly fluorescent) (Fig. 1c). Pseudoinclusions are oriented in the same direction within the resin layers (Fig. 1e,f). The thickness of the layers varies from 200 µm to 1 cm, although some layers appear thicker due to folding (Fig. 2a). Within a single piece, the layers may be more or less deformed. Figure 2a,b shows the contact between non-deformed and highly deformed layers. A few pseudoinclusion-rich layers tangentially cutting off previous layers have been found (Fig. 2c).

**Figure 3:**
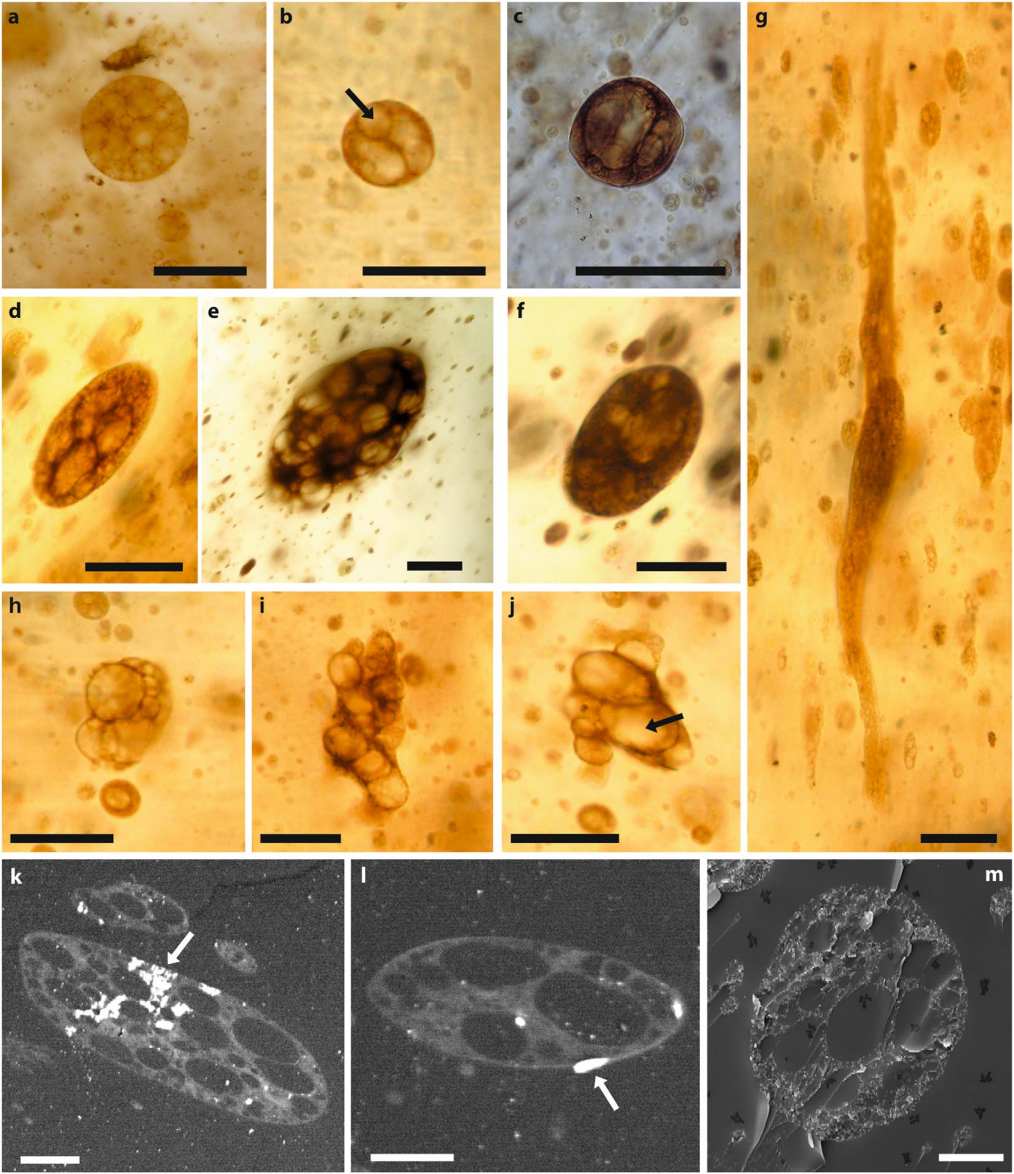
General aspect of pseudoinclusions showing the manner in which the phloem sap is present in the amber pieces, and the different degrees of deformation of the pseudoinclusions. Pseudoinclusions as seen with Light (**a‒j**) and Scanning Electron Microscopy (**k‒o**, **k** and **l** with BSE). (**a‒c**) Undeformed, nearly spherical pseudoinclusions (from preparations 18068, 18069 and 18711). (**d‒f**) Moderately deformed, ovoid pseudoinclusions (from preparation 18068). Note that the pseudoinclusion shown in (e) was analyzed with MRS, EMP and LSCM. (**g**) Large, highly deformed, elongate pseudoinclusion (from preparation 18068). (**h**) Undeformed pseudoinclusion of irregular morphology (from preparation 18711). (**i‒j**) Moderately deformed pseudoinclusion of irregular morphology (from preparation 18711). Arrows in (**b**) and (**j**) point to one of the light spheroids/ellipsoids (respectively) composing the pseudoinclusions and surrounded by dark matter. (**k‒l**) Moderately deformed, ovoid pseudoinclusions imaged at polished amber (from preparation 18068). Note the presence of electron-dense (mineralized) sub-inclusions located within the dark matter (arrows). (**m**) Moderately deformed, ovoid pseudoinclusion imaged on a broken amber surface (from sample 18614). Scale bars: 30 µm (**a,c**), 50 µm (**b,d,f,g,h,i,j**), 100 µm (**e**), 20 µm (**k,l,m**).

**Figure 4:**
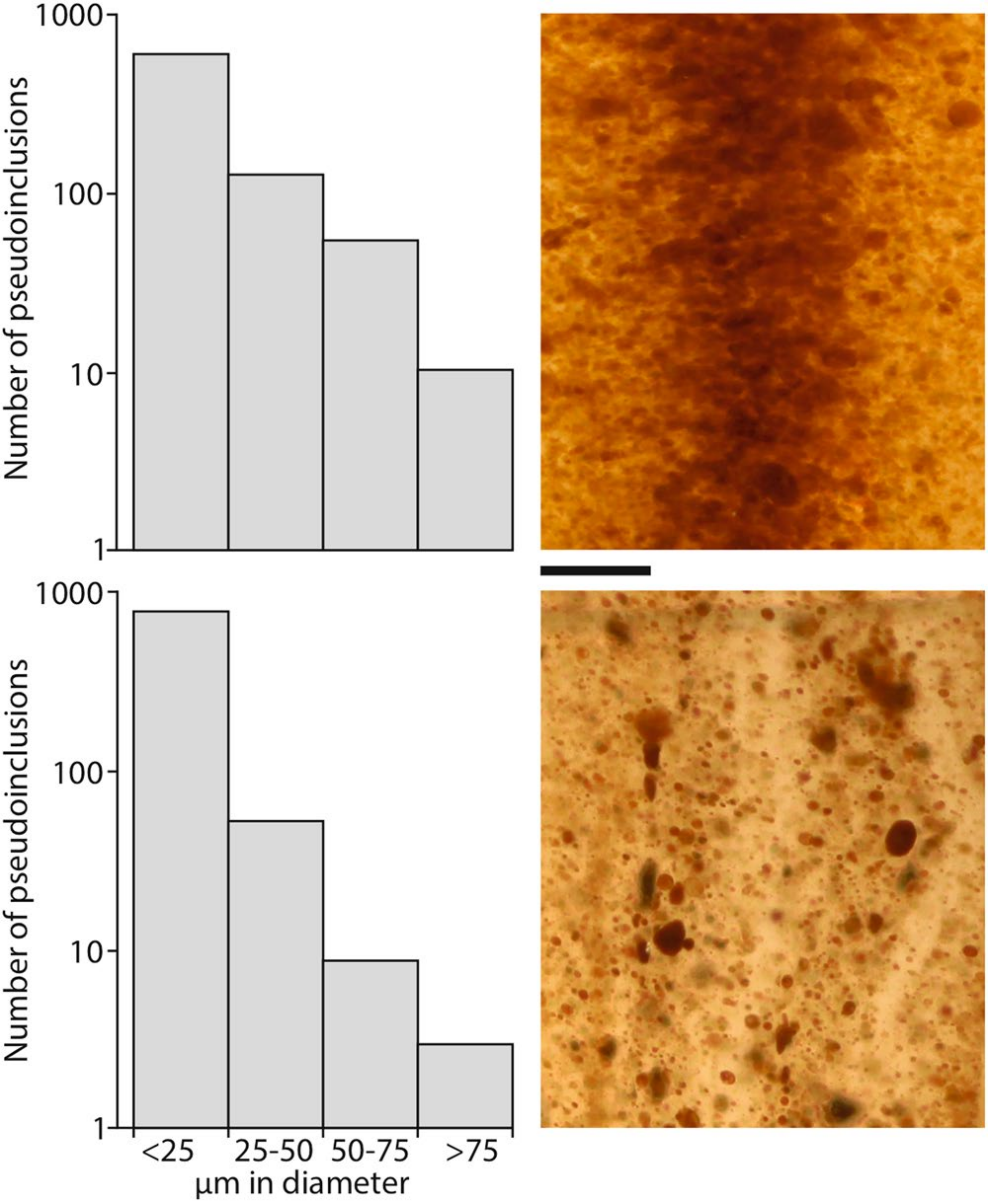
Histogram of size frequencies in two layers with high density of undeformed pseudoinclusions. Measurements taken from preparation 18068. Scale bar: 300 µm.

Nearly spherical pseudoinclusions, between 5 and 300 µm in diameter (Fig. 3a–c), are only found in non-deformed layers. The smallest pseudoinclusions (diameter <25 µm) are the most frequent and the largest ones are scarcer (diameter > 75 µm) (Fig. 4). In moderately deformed layers, the inclusions show ellipsoidal shapes (Fig. 3d–f), and when the layers are highly deformed, the pseudoinclusions are elongate, sometimes strongly so (Fig. 3g). Pseudoinclusions lack surface ornamentation (Fig. 3). Occasionally, they have irregular morphologies, resembling soapy foam, and show diverse degrees of deformation (Fig. 3h–j). Microstructurally, the pseudoinclusions are formed of light spheroids of different sizes (Fig. 3a–c) (or ellipsoids in moderately deformed inclusions; Fig. 3d–f) surrounded by dark matter. The volume of the dark matter compared to the volume occupied by the light spheroids/ellipsoids varies, although the proportion of the former is always lower. As expected, pseudoinclusions become darker as the proportion of dark matter increases. On polished surfaces under the SEM (BSE), this matter is more electron-dense than both the light spheroids/ellipsoids and the amber matrix, the latter two having the same appearance; in addition, the dark matter contains dense sub-inclusions with a high relative atomic weight, and appear highly bright (Fig. 3k,l). The vacuolated appearance of the pseudoinclusions is also apparent in broken amber surfaces using SEM (Fig. 3m).

### Spectroscopic data

**Figure 5:**
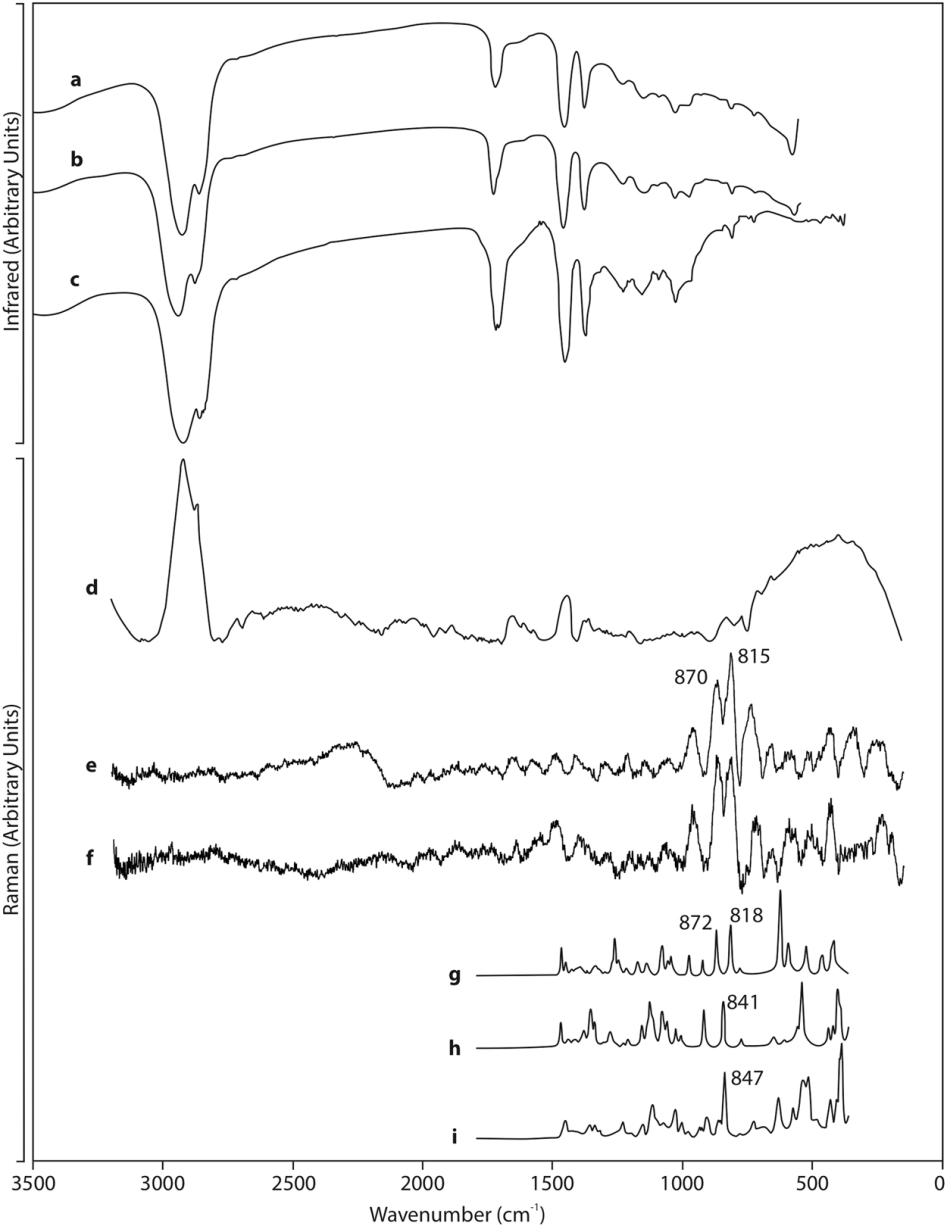
Measured spectroscopic profiles from Rábago/El Soplao amber, (**a‒c**) are FT-IR spectra and (**d‒f**) are MRS spectra; and MRS profiles of sugars (**g‒i**) taken from the literature. (**a**) Measured profile from a pseudoinclusion-rich amber fraction. (**b**) Measured profile from a pseudoinclusion-void amber fraction. (**c**) Orange amber from Rábago/El Soplao, extracted from^19^. (**d**) Measured profile from amber matrix. (**e‒f**) Measured profiles from the dark matter partly constituting the pseudoinclusions (each from a section belonging to different kidney-shaped pieces mounted on preparation 18068). (**g**) Profile of D-(-)-fructose; extracted from^33^. (**h**) Profile of b-D-glucose; extracted from^33^. (**i**) Profile of crystalline sucrose; extracted from^32^. Illustration created using CorelDRAW Graphics Suite X8 (www.coreldraw.com).

The Fourier Transform Infrared Spectroscopy (FT-IR) spectra obtained from a dark, pseudoinclusion-rich layer and a light, pseudoinclusion-void layer are practically identical, showing characteristic peaks at ~2950, ~1650 and ~1450 cm^−1^ (Fig. 5a,b). The Micro-Raman Spectroscopy (MRS) spectrum for the Rábago/El Soplao amber (Fig. 5d) shows the two most characteristic wavenumber regions at CH stretching vibrations between 2700 and 3100 cm^−1^ and skeletal functionality modes in the 1700–200 cm^−1^ region. Detailed band assignments for both FT-IR and MRS spectra are summarized in the literature^26,27^. The MRS spectra for the dark matter present in the pseudoinclusions from Rábago/El Soplao amber (preparation 18068) (Fig. 5e,f) show the two main peaks at 815 and 870 cm^−1^, in the 910–790 cm^−1^ region.

### Elemental data

**Figure 6:**
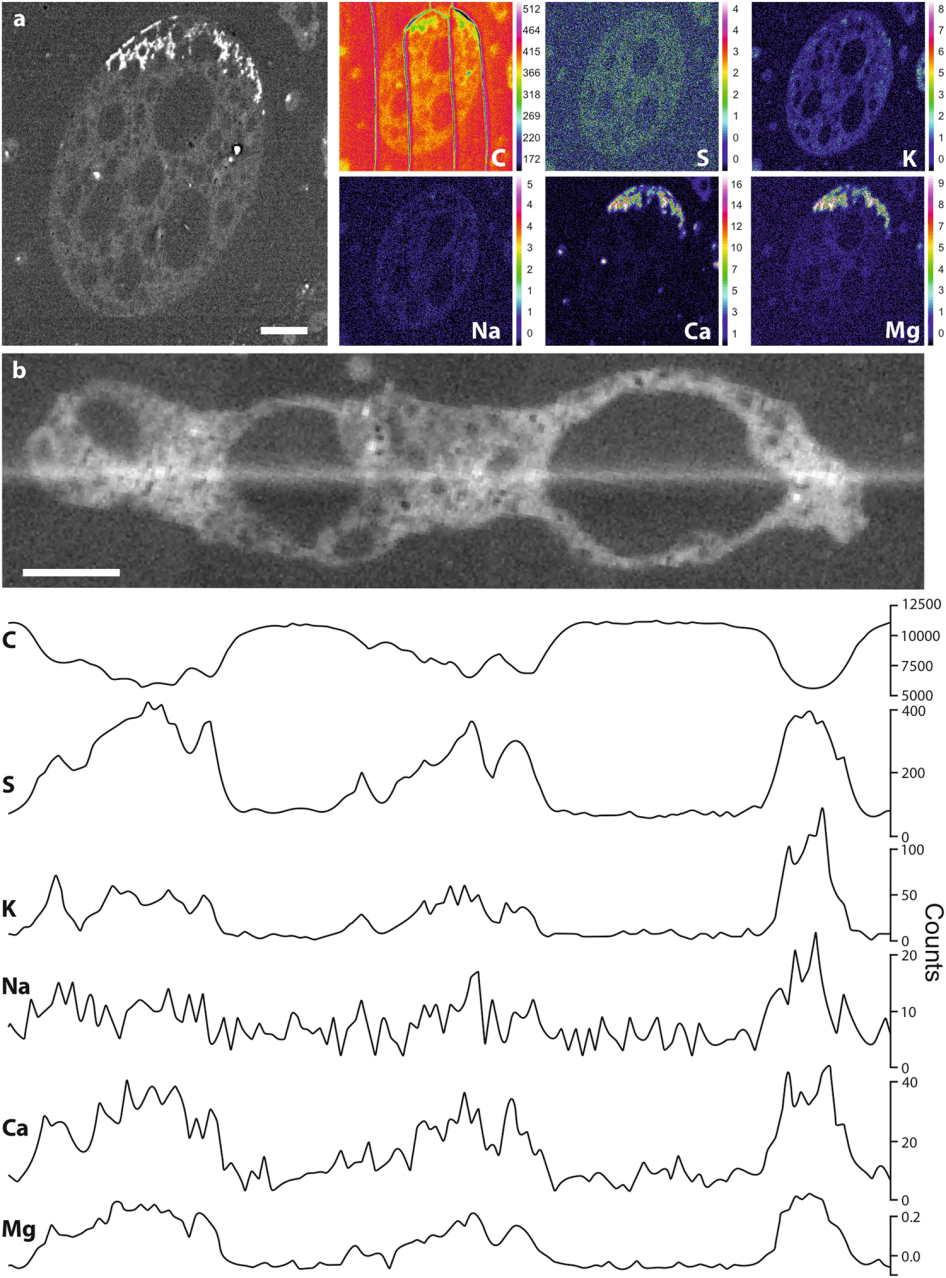
Chemical composition of pseudoinclusions. (**a**) Chemical composition map of a moderately deformed pseudoinclusion. Note how Ca and Mg are concentrated in mineralized sub-inclusions. Data obtained from a pseudoinclusion in preparation 18068 (left: BSE image) using EMP. Vertical lines in the C map are due to deterioration of the amber surface during scanning using the LDE1 multilayer diffracting crystal. (**b**) Chemical composition (lateral profile) of an irregular pseudoinclusion. Data obtained from a pseudoinclusion in preparation 18068 (top: BSE image) using EMP. Scale bars: 20 µm. Illustration created using Adobe Photoshop CS2, version 9.0 (www.adobe.com).

Electron Microprobe (EMP) analyses show that amber is namely composed of C, H, O and S (C~83; H~10; O~7; S~0.5 wt%; Table 1; preparation 18068). Among the inorganic elements analyzed (Ca, Mg, K, Na, Fe, Ti, P, Al and Cl), we have detected appreciable amounts of Ca, Cl, Mg, Al and K (values above the detection limit and ordered by concentration) (Table 1). In contrast, the dark matter compositionally differs from the amber in a noticeable enrichment in O and H and depletion in C (C~75; H~12; O~9 wt%). It also contains much more S (>3 wt%) and appreciable amounts of K, Cl, Ca, Mg, Na, and Al (values above the detection limit and ordered by concentration) (Table 1, Fig. 6).

**Table 1:** Chemical composition of amber, dark matter/phloem sap and mineralized sub-inclusions (aggregates).

Element	Amber	DM	C-Ca aggregates	C-Ca-Mg aggregates	Ca-P aggregates	C-K-Al aggregates
C	82.51	74.56	45.22	54.67	3.36	58.83
O	6.81	9.19	27.52	23.09	38.83	14.40
S	5194	33367	4367	4295	2963	11400
Ca	329	1427	194816	82050	365043	1440
Mg	157	957	641	26475	1193	1390
K	139	3556	750	795	1473	128140
Na	(34)	420	277	550	3147	6500
Fe	(185)	(595)	(850)	0	1230	(660)
Ti	(298)	(273)	643	390	565	0
P	(56)	(120)	1926	200	152373	0
Al	150	408	246	895	1840	28180
Cl	206	2231	n.d.	n.d.	n.d.	n.d.
Total	90.00	88.08	93.19	89.33	95.17	91.01
H*	10.00	11.92	n.d.	n.d.	n.d.	n.d.
n	27	20	7	2	3	1

All mean values obtained by EMP in three sections from different kidney-shaped pieces mounted on preparation 18068. C, O, H and Total data are in wt%, the rest in ppm. Hydrogen (H*) = 100-Total. Values for elements below the detection limit are shown between parentheses. Abbreviations: DM, dark matter/ phloem sap; n, number of measurements; n.d., not determined.

The electron-dense sub-inclusions present in most pseudoinclusions (Fig. 3k,l) are compositionally different from the dark matter. Whereas K is the most abundant cation in the dark matter (coinciding with the scarcity of mineralized K-rich aggregates), Ca is the most abundant cation in the sub-inclusions (Table 1). Although the chemical composition of the dark matter is quite homogeneous, that of the sub-inclusions is variable. Four types of sub-inclusions were recognized, with a Total between 89.33 and 95.17 wt% (Table 1, Fig. 6a): (a) calcium carbon sub-inclusions (Ca-C), which are the most common, and chiefly composed of carbon, oxygen and calcium (C~45; O~28; Ca~20 wt%), although they contain smaller amounts of S, Mg, K, Na, Ti, P and Al; (b) calcium magnesium carbon sub-inclusions (Ca-Mg-C) (C~55; O~23; Ca~8; Mg~3 wt%), which also contain smaller amounts of S, K, Na, Ti, P and Al (Table 1, Fig. 6a); (c) calcium phosphoric sub-inclusions (Ca-P) (O~39; Ca~37; P~15 wt%) with smaller amounts of S, Mg, K, Na, Fe, Ti and Al (here the C content may be partly enhanced by that of the surrounding amber, given that the size of some sub-inclusions is at the limit of the beam resolution); and (d) potassium aluminum carbon sub-inclusions (K-Al-C) (C~59; O~14; K~13; Al~3 wt%), which are the scarcest and also contain S (~1% wt%) and Na (~1 wt%), and smaller amounts of Ca, Mg and Fe.

### Fluorescence data

**Figure 7:**
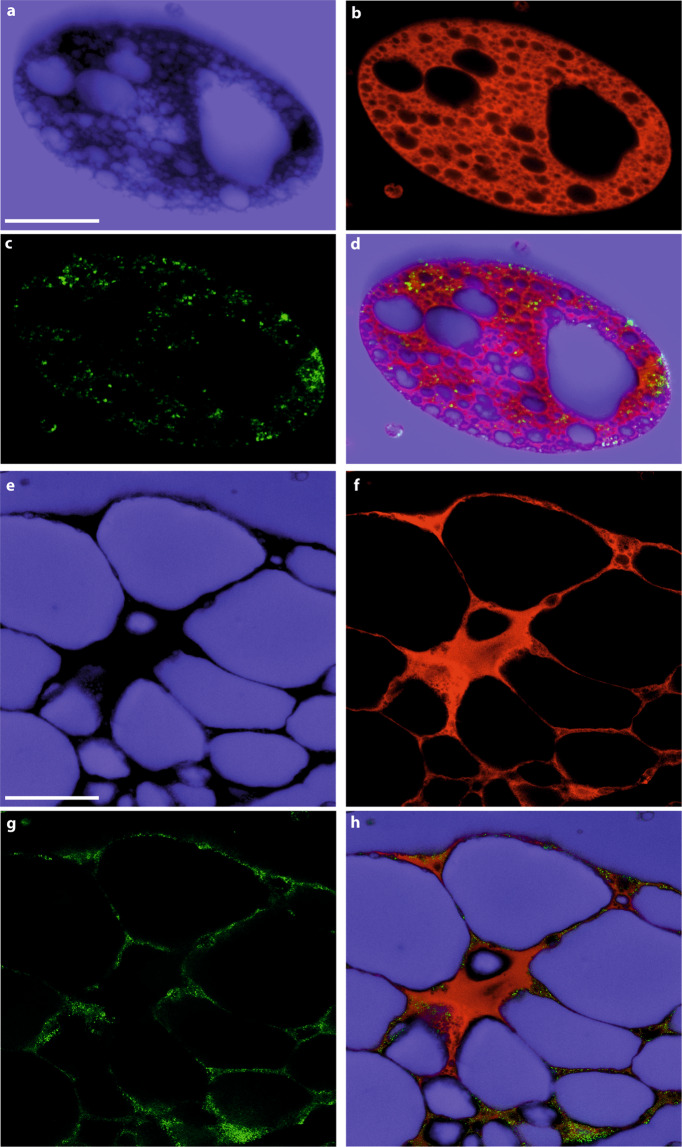
LSCM images of two pseudoinclusions at different magnification. Images taken from two sections obtained from different kidney-shaped pieces and mounted on preparation 18068. (**a**) Amber autofluorescence detected between 410 and 560 nm (max. at 437 nm) using 405 nm laser excitation. Note how under this excitation the dark matter (= fossilized phloem sap) is not fluorescent. (**b**) Dark matter/phloem sap autofluorescence detected between 570 and 700 nm (max. at 576 nm), using 552 nm laser excitation. (**c**) Reflection of mineralized sub-inclusions using 488 nm laser excitation. (**d**) Composite of (**a–c**) images by superimposing their pseudocolors. (**e**–**h**) Same as (**a–d**), respectively, but in a larger pseudoinclusion (ca. 200 ×300 µm; see Fig. 3e). Blue and red pseudocolors correspond to 405 and 552 nm laser excitation, respectively. Green pseudocolor in reflection mode appeared after excitation by 488 nm light. Scale bars: 15 µm (**a–d**), 50 µm (**e–h**).

Qualitatively, Laser-Scanning Confocal Microscopy (LSCM) images show an emission intensity under violet light excitation (405 nm) recorded in the blue spectral region (410–560 nm) (blue pseudocolor; Fig. 7a,e) for both the amber matrix and the light matter spheroids/ellipsoids of the pseudoinclusions. In contrast, although the dark matter of the pseudoinclusions does not fluoresce under violet or ultraviolet light (Fig. 7a,e), it does so using 552 nm laser excitation (red pseudocolor; Fig. 7b,f). In reflection mode, all sub-inclusions reflect the 488 nm laser (green pseudocolor; Fig. 7c,g). Figure 7d,h shows the combination of amber and dark matter emissions.

**Figure 8:**
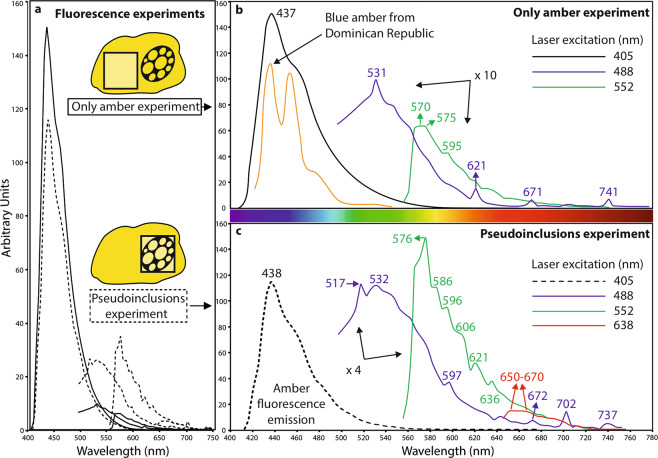
Fluorescence emission in Rábago/El Soplao amber. (**a**) Fluorescence emission spectra from the “only amber” (continuous line) and “pseudoinclusions” (dashed line) experiments (each from a section obtained from different kidney-shaped pieces and mounted on preparation 18068). (**b**) “Only amber experiment”. The two spectra obtained with 488 and 552 nm laser excitation have been magnified 10× for improved visualization (brown line, sowing blue amber Dominican Republic spectrum to comparison, from the literature^44^). (**c**) “Pseudoinclusions experiment” (area corresponding to Fig. 7e–h, from a pseudoinclusion shown in Fig. 3e). The three spectra obtained with 488, 552 and 638 nm laser excitation (dark matter/phloem sap fluorescence) have been magnified 4× for improved visualization. Illustration created using CorelDRAW Graphics Suite X8 (www.coreldraw.com).

LSCM measurements show a maximum emission intensity with 405 nm laser excitation for both the “only amber” and the “pseudoinclusions” experiments (see Materials and Methods section). It occurs at 437–438 nm (intensity of the maximum peak at 150 and 115 fluorescence arbitrary units, respectively), with one shoulder at approximately 460 nm (Fig. 8a). In the “only amber experiment”, the emission spectrum using 488 nm laser excitation shows signal in the 500 to 600 nm region, with a peak at 531 nm (10 fluorescence arbitrary units), and weaker peaks at 621, 671 and 741 nm (Fig. 8b). In addition, with 552 nm laser excitation the amber emits in the 565 to 700 nm range, with a weak peak at approximately 570 nm (6 fluorescence arbitrary units) and a shoulder at 595 nm (Fig. 8b). Lastly, in the “pseudoinclusions experiment”, the recorded fluorescence in green-yellow region is similar to that obtained in the “only amber experiment” using 488 and 552 nm laser excitation, but with some differences (Fig. 8c). The maximum emission with 488 nm of excitation occurs at 532 nm, although with another peak at 517 nm (27 fluorescence arbitrary units) and others with lower intensity at 597, 672, 702 (clearly absent in the “only amber experiment”) and 737 nm (Fig. 8c). With 552 nm laser excitation, the maximum fluorescence peak was recorded at 576 nm with numerous sub-peaks at 586, 586, 606, 621 and 636 nm. Finally, the 638 nm laser excitation produces a small emission in the red region, between 650 and 720 nm (Fig. 8c).

### Mass spectrometric data

The Electrospray Ionization Mass Spectrometry (ESI-MS) spectrum from the analyzed pseudoinclusion-rich dark fraction shows two characteristic peaks at m/z 188.65 and m/z 359.23 (Fig. S1), which are absent in the analyzed pseudoinclusion-void light fraction. The molecular mass corresponding to the peak at m/z 188.65 is 374 g/mol, assuming the proton as an ionization mechanism, giving (M + 2H/2)^2+^ . Likewise, the molecular mass corresponding to the peak at m/z 359 is 761 g/mol. The tandem mass spectrometry (MSMS) fragmentation spectrum of the peak at m/z 359 shows two consecutive water losses at m/z 350 and m/z 341, also with 2+ charge.

## Discussion

From the morphological standpoint, the undeformed pseudoinclusions^12,16^ (Fig. 3a‒c) resemble emulsion droplets from double emulsions^28,29^. If an emulsion is a mixture of immiscible liquids, a double emulsion is an emulsion of emulsions, where droplets of the dispersed phase themselves contain smaller droplets^28^. More specifically, undeformed pseudoinclusions are most similar to the type C double emulsion droplets described by some authors, which contain a high number of the smaller, internal droplets^29^. Double emulsions have been intensively studied for decades due to their widespread use in the pharmaceutical, cosmetic, oil, agricultural and food industries^28^, and their manufacturing and demulsification are still a hot topic of research. The most common types of double emulsions are water-in-oil-in-water (w/o/w) and oil-in-water-in-oil emulsions (o/w/o). In the latter, a hydrophilic phase separates two hydrophobic ones: the primary dispersed phase in the inner emulsion is hydrophobic, the secondary dispersed phase in the outer emulsion (the intermediate phase) is hydrophilic (polar), and the final continuous phase is, again, hydrophobic^28^.

Unlike in the stalactite-shaped amber pieces, the inner layering of which is related to successive resin flows that covered previous ones dried by exposure to aerial conditions (e.g., sunlight, wind^3^), the banding pattern in the kidney-shaped amber pieces can be explained by a different process. In each of these pieces, most of the new resin inputs became injected into a previously emitted resin accumulation^22^. This circumstance is shown by the abundant pseudoinclusion-bearing dark layers bent by ductile deformation (Fig. 2a,b) and the altered morphology of the pseudoinclusions themselves, which were stretched to different degrees in the same direction of the resin deformation (Fig. 3a–g). Thus, although the original morphology of most of the pseudoinclusions was nearly spherical, in deformed layers they did not recover such shape because of resin viscosity. However, the resin must have remained relatively fresh to enable the injection of new inputs. In that regard, resin emission in unexposed conditions, such as on roots or in trunk pockets, would have slowed down the volatile loss and resin hardening process^22^, increasing the time in which resin remained ductilely deformable and, thus, favoring the accumulation of pseudoinclusion-bearing dark layers. Although most resin inputs were emitted into ductile resin masses, some inputs intruded into hardened resin bodies, in-filling small fractures (Fig. 2c). The dark coloration of the pseudoinclusions is not deemed to be diagenetic in origin, at least not entirely, as these inclusions present in modern resin are already darkened^16^.

During the Cretaceous, fungal mycelia commonly consumed the fresh, kidney shaped resin emissions from their external surface inwards, creating light brown to opaque cortices^22^. Note that the identification of this Cretaceous filamentous, heterotrophic microorganism is currently under debate^30^. In the studied kidney-shaped amber pieces, the mycelia grew towards the core of the resin emissions, preferably following the layers exhibiting a higher density of pseudoinclusions (Fig. 1d–g). This strongly suggests that the pseudoinclusions were more nutritious for the fungi than the resin alone. Among the substances secreted by plants, phloem sap is rich in nutrients and generally free of deterrent substances and toxins, and has been shown to allow bacterial growth and multiplication^31^.

The overall quantity of dark matter compared to that of amber in the pseudoinclusion-rich layers is low, as indicated by the virtually identical FT-IR spectra between the analyzed samples (Fig. 5a,b) and the similarity between both spectra and that of raw amber without pseudoinclusions from Rábago/El Soplao^19^ (Fig. 5c). On the other hand, the micro-Raman spectrum obtained for the Rábago/El Soplao amber (Fig. 5d) is similar to that of amber or resin from any age due to their common chemical functional groups^26^. Remarkably, the main peaks in the 1200–700 cm^−1^ range (Fig. 5e,f) shown by the two micro-Raman spectra obtained from the dark matter partly constituting the pseudoinclusions coincide with those characteristic for sugars^32,33^ (Fig. 5g‒i), especially in the regions of δ(COH), δ(CCH) and δ(OCH) side group deformations^31^. Although these results do not allow to identify the exact molecular composition of the pseudoinclusion dark matter, it likely contains sugar residues that degraded during diagenesis. In the vascular system of terrestrial plants, whereas the xylem transports water and mineral nutrients taken up by the roots from the soil to the aerial part of the plant, the major role of the phloem is to transport the photosynthates from a photosynthetically active source to sink tissues^34^. Xylem sap contains carboxylates, hormones, amino acids, peptides and proteins; phloem sap is rich in nutrients and contains mostly sugars and amino acids, as well as organic acids, vitamins and inorganic ions^31,35^. Although sucrose is widely believed to be the most predominant sugar transported by the phloem in most plants, other sugars such as hexoses (including fructose and glucose) have been found in significant proportions in the phloem sap of certain plants^31^, yet their ability to be transported in the phloem remains controversial^36^. The data obtained using ESI-MS (Fig. S1) show the presence of molecules with molecular masses of 374 and 716 g/mol, which could indicate transformation or polymerization processes of the original sugar. In any case, obtaining two consecutive water losses during MSMS fragmentation of 359 peak (m/z 350 and m/z 341) suggests that the substance of molecular mass 716 g/mol is polar and contains at least two OH groups^37^.

The organic chemical composition of the analyzed amber sample from Rábago/El Soplao (C, O and S; Table 1) is similar to that of other Cretaceous and Cenozoic fossil resins^38^. Regarding trace (inorganic) elements, results from the analyzed sample are coincident with previous analyses of fossil resins that show that Ca is the most abundant trace element in the amber, with presence of other elements in minor proportion^39^. More importantly, the chemical composition of the dark matter significantly differs from that of the amber matrix containing the pseudoinclusions (Table 1, Fig. 6b). Only Na and K were previously identified in pseudoinclusions using ToF-SIMS^16^. Remarkably, most of the inorganic cations present in the dark matter occur as mineralized sub-inclusions, which become apparent due to their electron-dense appearance (Fig. 3k,l), variable elemental composition and distribution within the pseudoinclusion (Table 1, Fig. 6), as well as fluorescence reflectivity (green pseudocolor) (Fig. 7c,g). Even though the minute size of these mineralized aggregates has prevented a more detailed characterization, their mineral composition must be similar to calcite (C-Ca aggregates), dolomite (C-Ca-Mg aggregates) and apatite (Ca-P aggregates). The fact that the stoichiometric relationships of the elements are not as expected is probably due to contamination of the dark matter matrix that houses the mineralized aggregates. The most plausible scenario is that these mineralized sub-inclusions were formed by concentration of most of the ions originally dissolved in the dark matter during diagenesis. As a result, the inorganic chemical composition of the dark matter itself (Table 1) is not fully representative of the composition of the original fluid. Although our results do not show the presence of pyrite in the samples (note also that almost all Fe contents are below the detection limit), this mineral was abundantly detected within pseudoinclusions in previous studies^10,11^. These studies most probably analyzed different types of amber pieces (not necessarily kidney-shaped) or pseudoinclusions that had a more superficial position within the amber pieces, and were therefore more susceptible to cracks favoring typical allochthonous iron and sulfur infiltration.

All the inorganic elements found in the dark matter and the mineralized sub-inclusions (Table 1), except Ti (the latter present in negligible amounts or nearly so), are found in the phloem sap of extant plants, with concentrations that vary depending on the plant species and environmental conditions:^35,40^ Ca is an important element for phloem physiology and signaling; K promotes the load of sugar in leaves and is required for the transmission of electric signals; P is involved in the control of the phloem sap’s pH; Mg, Na and Cl contribute to the leaf’s solute balance; Fe is one of the micronutrients involved in carrying the sap; and S is a critical macronutrient for plant growth related to the production of essential amino acids^35^. Although Al is toxic for most plants, for some it appears to be beneficial or necessary, and could be transported by the phloem sap^41^. The presence of traces of these inorganic elements in Cretaceous amber has been interpreted as a consequence of the interaction between the resin and the sap of the same tree, and thus indirectly related to soil-water composition^39^. In that regard, aqueous inclusion droplets from Baltic amber^42^ could represent inclusions of sap, since they contain Na, K, Ca, Mg, S and Cl. These aqueous inclusions were primarily interpreted as being derived from water splashed from a saline environment into the resin, yet the authors also suggested that the sap of the producing tree could have also been involved in the formation of these inclusions due to presence of ammonium and acetate ions^42^. Lastly, it is important to note that, although amber is a relatively permeable substance, most likely the detected inorganic elements are not contamination originated from the amber-bearing rock during diagenesis. First, the amber pieces analyzed lack internal cracks or any external signs of alteration. Moreover, if contamination had occurred, the obvious compositional difference between the amber and the dark matter would be less likely.

Some ambers exhibit a characteristic bluish to violet color when exposed to long-wave ultraviolet light, including sunlight. This type of amber is known as “blue amber” and, in the Cenozoic, it is known from the Eocene of the Far East of Russia^43^, and the Miocene from the Dominican Republic and Indonesia^27,44^. A fluorescence microscopy study of Dominican amber showed that its maximum emission (449 nm) is very similar to that of perylene (443 nm^44^); FT-IR and gas chromatography-mass spectrometry studies did not find a distinct signature of this compound in Russian blue amber, even though its autofluorescence is similar to that of the Dominican Republic amber^43,45^. The only Cretaceous blue amber reported to date is extracted from the Rábago/El Soplao deposit^19^. The main autofluorescence from this amber, detected in the blue spectral region (peaking at 437 nm) after 405 nm laser excitation (Fig. 8b), is notably similar to that of the perylene. However, neither FT-IR nor gas chromatography-mass spectrometry analyses of Rábago/El Soplao blue amber found the presence of perylene; instead, the azulene derivative guaiazulene was detected, which could be responsible for the blue fluorescence^19,20^. The intense blue fluorescence exhibited after violet light excitation by both the amber matrix and the light matter spheroids/ellipsoids that give the pseudoinclusions their vacuolated appearance (Fig. 7a,e) indicates that the light matter represents amber. The possibility that the light matter could be empty is ruled out, because it is clearly filled in exposed amber surfaces and has the same appearance as the amber matrix under SEM (Fig. 3k‒m). The lack of blue fluorescence in the dark matter (Fig. 7) demonstrates that it does not contain azulene-derived compounds, at least in significant amounts. Thus, the blue emission (peaking at 438 nm) detected in the “pseudoinclusions experiment” is identical to the signal present in the “only amber experiment” (yet slightly weaker) (Fig. 8c) and must correspond to the emission of the amber, both from the matrix containing the pseudoinclusions and from the “vacuolated” amber within the pseudoinclusions.

The dark matter emits in the green-red, yellow-orange, and red regions of the spectrum, using 488, 522 and 638 nm laser excitation, respectively (Fig. 8). Although amber also emits in the same regions using the same laser excitations, the green-red emission of the dark matter is much more intense than that of the amber (approximately three times the intensity), and it shows a somewhat different fluorescence profile (Fig. 8). On the one hand, the presence of autofluorescent substances in the amber and the dark matter emitting in the same regions strengthens the idea that both fluids were produced and emitted by the same tree individual^14,15^. It has been suggested, based on analyses of inorganic trace elements present in French Cretaceous amber, that a certain compositional relationship existed between the resin and the sap^39^. On the other hand, the fluorescence emission in the green-red region of the spectrum for both the dark matter and the amber can be interpreted as content of plant pigments. Although the data obtained are insufficient for a detailed assessment of the fluorescent substances, we suggest the presence of residues of carotenoids (emitting at the 520–580 nm region of the spectrum^46^), anthocyanins (emitting at 520–610 nm region^46^) and chlorophylls (emitting in the red region^47^). These three pigment types are the most important for vascular plants^48^. Anthocyanins are soluble in water^48^ and they are found in sap (probably having a deterring effect on phloem sap feeders)^49^. In contrast, carotenoids and chlorophylls are not soluble in water^48^. However, carotenoids are known to be hydrosoluble when forming complexes with proteins and sugars^48^, both of which are abundant in phloem sap^35^. In addition, carotenoids have been identified in the wood of various tree species^50^, suggesting that the *in situ* formation of carotenoids in the living cells occurs in the sapwood^48^. Regarding chlorophylls, minor residues could have been incorporated (traumatically?) into the dark matter from photosynthetic tissues, which could explain the obtained low-intensity fluorescent signal.

The sum of the evidence presented here proves that the pseudoinclusions, common in Cretaceous ambers and originally misidentified as microbes, are composed of amber equivalent to the amber matrix and of dark matter containing inorganic elements and organic compounds, the latter likely representing diagenetically altered remnants of sugars and plant pigments. Morphologically, taphonomically and compositionally, the dark matter can be most conservatively interpreted as phloem sap produced by the same resin-producing tree. This is the first time that fossilized sap has been recognized and characterized. Moreover, the pseudoinclusion-rich, kidney-shaped amber pieces represent fossilized, resin-in-sap-in-resin double emulsions, i.e., polydispersed resin within droplets of sap, the latter being polydispersed in a resin matrix themselves. A resin-in-sap-in-resin double emulsion is comparable with an oil-in-water-in-oil double emulsion, in that the outer continuous phase is hydrophobic, and the phase dispersed within is hydrophilic and, in turn, contains the hydrophobic phase dispersed within. The double emulsion was formed when the ducts through which the phloem sap circulated were affected by tree damage that promoted resin exudation, and variable quantities of phloem sap mixed with fresh resin. As both immiscible liquids became mechanically joined and were intruded into a budding kidney-shaped resin body, a variable number and size of proto-pseudoinclusions (in other words, resin-in-sap emulsion droplets) were created. As resin injection continued, the previously secreted resin layers suffered a ductile deformation that distorted the shape of the resin-in-sap emulsion droplets within. The differential content of such droplets in suspension caused dark-light banding patterns to be formed. While the resin was still soft, the phloem sap embedded in the resin was opportunistically and preferentially consumed by Cretaceous resinicolous fungi. The sugar originally contained in the phloem sap could have polymerized during diagenesis, forming polar molecules of a higher molecular weight than sugars. Diagenetically, an important part of the dissolved ions originally present in the phloem sap were selectively concentrated as aggregates, forming carbonate and phosphate minerals. The data presented here provide definitive evidence for ruling out the microbiological nature of the protist-/microbe-like inclusions, as was previously advocated based on morphological, distributional and ToF-SIMS analyses^16^. Interestingly, although pseudoinclusions have occasionally been described from Cenozoic ambers, the pseudoinclusion-bearing dark layers typical of kidney-shaped amber pieces from Cretaceous ambers worldwide have not. For that reason, it is possible that only small amounts of sap were incorporated into fresh resin by resin-producing plants during the Cenozoic. This could be due to multiple factors affecting resin production and secretion, although that issue is beyond the scope of this study. In any event, the study of the fossilized, resin-in-sap-in-resin double emulsions offers a promising new tool to characterize diverse amber types from different ages and deposits worldwide. Specifically, bringing the knowledge that industry has gained on double emulsions to the paleobiology and geochemistry of organic resins can shed light on many significant subjects. These include the identification of the resinous plant sources, addressing plant physiology in deep time (e.g., responses to pathogen-mediated diseases), inferring paleoenvironmental parameters, determining taphonomic features of the ancient resin production (location, timing, viscosity), or understanding physicochemical transformations during diagenesis.

## Materials and Methods

A total of 32 sections, 0.5–1 mm thick, and ranging in size from 2 × 3 mm to 15 × 30 mm were prepared from different amber pieces from the Rábago/El Soplao outcrop. Although these were mostly fragmentary when unearthed, they were attributed to kidney-shaped pieces based on their irregular morphology (not stalactite- or flow-like), surface characteristics^19,22^, and their inner banding, which is consistent with complete kidney-shaped amber pieces (see below). From the total number of sections, 22 were mounted together on four glass slides using epoxy resin, i.e., preparations 18068 (ten sections), 18069 (six sections), 18070 (five sections), and 18711 (one section). A full section of the same thickness (preparation 18067), 12 × 8 cm, was taken from a complete kidney-shaped amber piece and prepared on a glass slide; this sample was previously embedded in epoxy resin for consolidation before cutting. The remaining nine sections, 18071‒18076 and 18614‒18616, were unmounted. All the studied samples and preparations are deposited at the Museo Geominero (Instituto Geológico y Minero de España, Madrid, Spain).

### Light and scanning electron microscopy (SEM)

All samples were examined using a Leica DMLP and an Olympus BX51 optical microscopes. The size distribution of pseudoinclusions in two non-deformed layers from two amber sections (mounted on preparation 18068) was calculated in sectors of 1.45 ×1.20 mm using the computer software FIJI for Windows (67 bits; https://fiji.sc/). Gold coated, freshly broken surfaces of samples 18614, 18615 and 18616 were observed using a JEOL 6400 Scanning Electron Microscope (SEM) equipped with an energy-dispersive X-ray microanalyzer and a back-scattered electron detector (BSE) at the Centro Nacional de Microscopía Electrónica (CNME) of the Universidad Complutense de Madrid (UCM).

### Fourier transform infrared spectroscopy (FT-IR)

Two amber fractions from different kidney-shaped amber pieces that had been washed in order to remove surface finger grease and dust were selected: a pseudoinclusion-rich, dark fraction, and pseudoinclusion-void, light fraction. From them, solid amber samples were pulverized and analyzed using the KBr tablet technique (1 wt% sample) on a BRUKER IFS66v spectrometer, at the Servicio Interdepartamental de Investigación (SIdI) of the Universidad Autónoma de Madrid (UAM), Spain. A total of 250 scans were taken to improve the signal to noise ratio in the 7000–550 cm^−1^ range. The normal resolution was 4 cm^−1^.

### Micro-Raman Spectroscopy (MRS)

Two sections obtained from different kidney-shaped amber pieces and mounted together on preparation 18068 were analyzed. Sections underwent cleaning with distilled water and compressed air to remove surface grease and dust. The micro-Raman spectra of the spot samples were performed with a Thermo-Fischer DXR Raman Microscope. The system consists of an Olympus BX-RLA2 Microscope, a CCD (1024 × 256 pixels) detector, a monitored XY stage, an auto-focus, and microscope objectives Olympus UIS2 series, all controlled with the software OMNIC 1.0 (https://www.thermofisher.com/), and is located at the Laboratorio de Técnicas No Destructivas of the Museo Nacional de Ciencias Naturales (Consejo Superior de Investigaciones Científicas), Madrid. Light at 780 nm of a frequency-doubled solid laser (maximum power 22 mW) was used for excitation. The average spectral resolution in the Raman shift ranging from 100 to 3600 cm^−1^ was 4 cm^−1^, i.e., grating 900 lines/mm and 2 µm spot sizes. The system was operated under OMNIC 1.0 software fitting working conditions such as pinhole aperture of 25 µm, bleaching time 30 s and four exposures of 10 s each on average. Even though a total of eight spectra were obtained from the dark matter within pseudoinclusions (with five pseudoinclusions with diameters close to 300 µm; Fig. 3e), only two spectra from two different inclusions did not show interference by the light spheroids/ellipsoids (amber). The laser substantially deteriorated the sample’s surface.

### Electron microprobe analysis (EMP)

The sections analyzed with this technique were the same two utilized for the MRS study, plus an additional section (all three mounted on preparation 18068) (Fig. 3e). Sections were polished a few tens of microns to access areas unaltered by the Raman laser and surfaces were cleaned using distilled water. Chemical analyses of amber, pseudoinclusions and mineralized sub-inclusions present within the latter were performed on chrome-coated polished sections using a JEOL JXA 8900 Electron Microprobe (EMP), operating at 15 kV, 20 nA, and 5–1 µm beam diameter, at the CNME (UCM). Detection limits are approximately 1000 ppm for C, 2200 ppm for O, 150 ppm for S, 100 ppm for Ca, 90 ppm for Mg, 120 ppm for K, 100 ppm for Na, 1000 ppm for Fe, 320 ppm for Ti, 150 ppm for P, 130 ppm for Al, and 80 ppm for Cl. Carbon and oxygen were measured using a LDE1 multilayer diffracting crystal, yielding a statistical precision similar to heavier elements. Measurements of N with the same channel were unsuccessful because amber deteriorated rapidly when the voltage necessary for proper measurements was applied. The content in H was estimated by subtracting the average analytic Total from 100. Only 60 out of the 207 EMP measurements taken from the pseudoinclusion constituents (i.e., light spheroids/ellipsoids, dark matter, and mineralized sub-inclusions) were used as the remaining showed interference with one another due to their small size. The amber surface deteriorated rapidly due to the emission of energy produced by the BSE setting.

### Laser-scanning confocal microscopy (LSCM)

The sections analyzed for this technique were the same two utilized for the MRS and EMP studies (mounted on preparation 18068) and came from different kidney-shaped amber pieces. The sections were further polished to access areas unaltered by the previously used techniques, and their surfaces were cleaned again with distilled water. LSCM was used to study the morphology of the pseudoinclusions, the distribution of mineralized sub-inclusions, and to calculate the emission spectra of both the amber matrix and the dark matter. We carried out two experiments at room temperature (20–22 °C) using a Leica SP-2 AOBS Confocal Microscope at the Centro de Apoyo a la Investigación of the UCM: a sample of amber lacking pseudoinclusions (named “only amber experiment”) and a sample containing pseudoinclusions but also including amber (named “pseudoinclusions experiment”). For the latter, a 180 × 180 µm area within a large pseudoinclusion (ca. 200 × 300 µm in diameter; Fig. 3e) was selected to obtain the emission wavelengths. Image analysis was performed with the computer software FIJI. Images were taken at different focal depths in two different modes: fluorescence, where the microscope collects the signal that is emitted from the sample through autofluorescence, and reflection, where the microscope collects the laser signal that is reflected by the sample. In fluorescence mode, emission excitation was done using diode lasers operating at 405, 488, 552 and 638 nm (although the latter wavelength was only used for the “pseudoinclusions experiment”). In reflection mode, the diode laser operating at 488 nm and 552 nm was used to distinguish the mineralized sub-inclusions within pseudoinclusions.

### Electrospray ionization mass spectrometry (ESI-MS)

The same pseudoinclusion-rich, dark fraction and pseudoinclusion-void, light fraction used in the FT-IR analysis were used here. Mass spectra were obtained using an ultra-high-resolution QTOF instrument (QSTAR Pulsar i, ABSciex) at the SIdI (UAM). The extraction was carried out with water and the samples were dissolved in methanol for their ionization and introduced in the mass spectrometer to 20 ul/min with an infusion pump. Electrospray ionization source in positive mode was used for all the analyses and the parameters were adjusted as follows: capillary voltage 5500 V, focusing Potential 210 V, declustering potential 30 V. Nitrogen was used as nebulizer gas (pressure of 10 Bar) and the scans of MS spectra were conducted in the m/z range of 50 to 2000. For accurate high resolution mass spectrometry (HRMS) internal calibration was performed after analysis. A tandem mass spectrometry (MSMS) fragmentation spectrum of m/z 359.23 peak has been performed to determine parts of the molecule. The accurate masses obtained were processed using the elemental composition calculator incorporated in the Data Analysis Software (ABSciex) (https://sciex.com/products/software/analyst-software).

## Supplementary information

Supplementary information.
