# Supplementary material for: Phloem sap in Cretaceous ambers as abundant double emulsions preserving organic and inorganic residues
Source: Sci Rep. 2020 Jun 16;10:9751. doi: 10.1038/s41598-020-66631-4 (PMC7297994; doi:10.1038/s41598-020-66631-4)
Supplement: Supplementary file 1 — Supplementary information. [file 41598_2020_66631_MOESM1_ESM.docx]

Supplementary Information for

**Phloem sap in Cretaceous ambers as abundant double emulsions preserving organic and inorganic residues**

Rafael Pablo Lozano, Ricardo Pérez-de la Fuente, Eduardo Barrón, Ana Rodrigo, José Luis Viejo & Enrique Peñalver


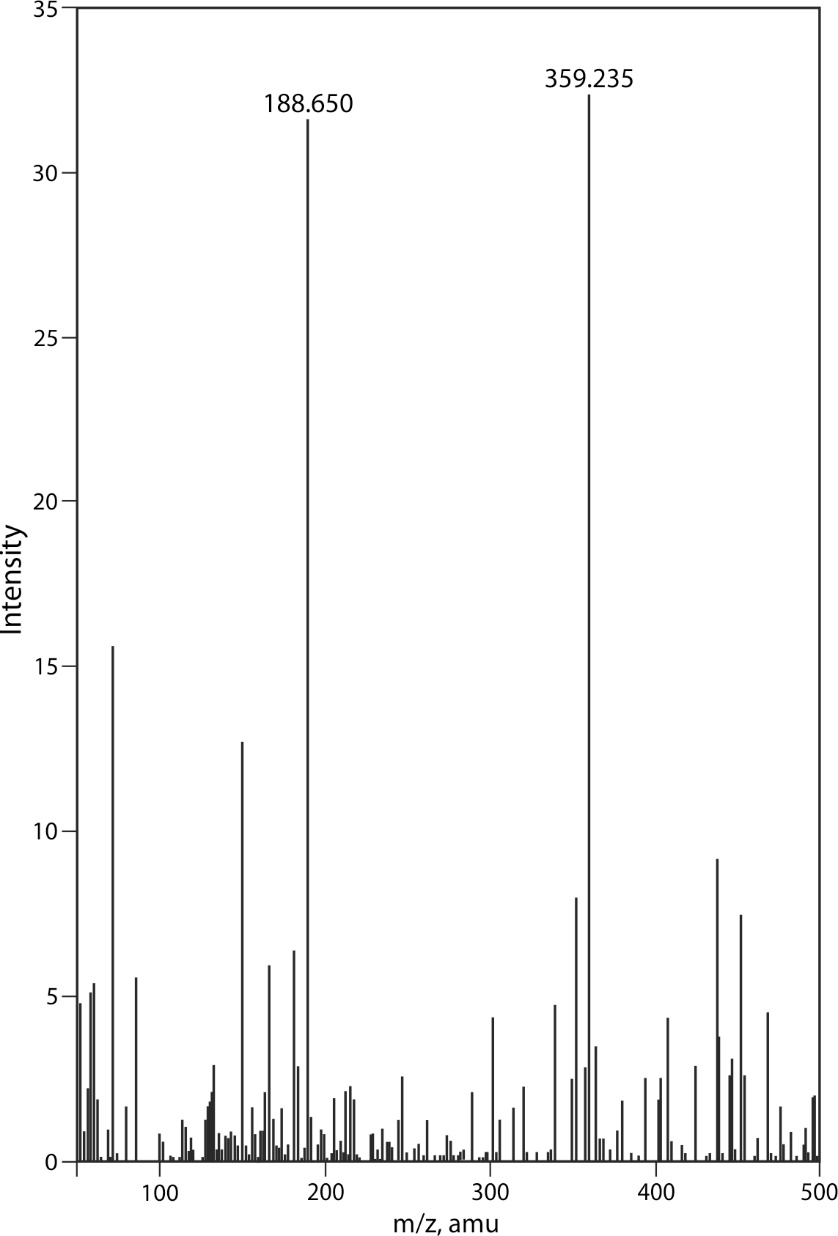


**Fig. S1.** Electrospray Ionization Mass Spectrometry (ESI-MS) spectrum from a pseudoinclusion-rich dark fraction. Note the two characteristic peaks at m/z 188.650 (374 g/mol) and m/z 359.235 (761 g/mol).
